# A Comparison of Old and New Style Attachments in Fixed Bridges

**Published:** 1909-12-15

**Authors:** R. B. Howell

**Affiliations:** Ann Arbor


### "A COMPARISON OF OLD AND NEW STYLE ATTACHMENTS IN FIXED BRIDGES."

BY R. B. HOWELL, D.D.S., ANN ARBOR.

Read before the Michigan Second District Dental Society, Nov. 2nd, 1909.

It is fortunate in the selection of a subject along any practical line that there is never a dearth of questions to discuss, and the problem is never what can but what shall one write about. Any line of work which depends entirely upon the creative and manipulative ability of the operator is certain to present some opportunities for a difference of opinion. So the task of the writer in preparing a paper on this occasion and subject is an easy one. In fact there are so many phases of the subject he would like to have discussed that he experienced difficulty in making a choice.

Believing, however, that the chief function of a paper is to bring out a liberal discussion we have chosen this particular phase of the subject feeling assured that every one present will have something to add.

It is probably believed by all of us, that the prevalent method in the application of the principles in Bridge Work by our profession are entirely too transcendental. Man is such a creature of habit, that in this, as in other things he is too prone to adhere to those methods which were early taught and then impressed upon him as infallible. Of course they may be in many cases, but when misapplied through lack of proper consideration they may also become most fallacious. No empirical method can be followed. The tube and split post is not always the best attachment for removable bridges, nor is the band and dowel the *sine qua non* in attachments for fixed bridges, any more than the old solid rubber tire of a bicycle would be adaptable to an automobile.

The purpose of this paper therefore is to bring out more thought and display of intelligence in the application of the principles which govern attachments in fixed bridges. The subject chosen is simply one of many which could be considered for this purpose.

It is not necessary to discuss in detail the *importance* of the correct preparation of abutments and perfect construction of the attachments to assure success in bridge work, suffice it to say that just as "no chain is stronger than its weakest link" so no bridge is stronger than its weakest attachment or dummy. And as the perfect construction of a foundation is most necessary to the permanency of an edifice, so the correct preparation of the abutments and restoration thereon of the best attachments possible, both in principle and mechanical structure, is the thing to be absolutely obtained to insure the desired stability and longevity of the bridge.

In order to approach this subject in a sowewhat systematic manner, let it be taken for granted that a case for bridge-work is presented. Now what is the duty of an up-to-date practitioner of Dental Prosthesis, what is there for his consideration? In the first place he should keep in mind what his branch of the profession suggests, that he is by an artificial method endeavoring to restore a lost function; and it might be added one of the most important functions in the human body, as mastication is the first step in the process of body nutrition. The proper preparation of food for deglutition depends then upon the restoration in that mouth of a masticatory apparatus as ideal and natural for that mouth as is possible. The completed structures should in no way interfere with the normal physiological condition found in that mouth; there should be nothing irritating to the soft tissues and the physiological individuality of each tooth used as an abutment should be retained if possible, in fact, both of these conditions may sometimes be improved upon. It should be perfect in mechanical structure (both attachments and dummies), strong enough to withstand all force of mastication, of a material thoroughly compatible with its environments; it should be ideal from the anatomical aspect,—perfect occlusion and alignment, the exact form should be restored and last it should be natural in appearance, in other words hide its artificiality.

The place of attachments in this ideal structure can easily be seen and it can be stated very positively that the success or failure of this structure will depend upon them.

In the discussion of the requirements for attachments it can best be considered under these four heads: 1. Physiological. 2. Mechanical. 3. Anatomical. 4. Esthetic.

As to the Physiological requirements, the first thing to do in the treatment of the case is by a series of thorough prophylactic treatments put the mouth in a normal, healthy condition. Almost 90 times out of a 100 cases for bridge work require this treatment, simply because most of the teeth have not been performing their function and just as atrophy always follows lack of use, so the soft tissues have become diseased. After this has been accomplished the next step is to obtain good plaster impressions and pour models from the same and then mount the models upon an articulator which at least *endeavors* to imitate the migrations of the mandible, so that the articulating movements can be ascertained. From these models a diagnosis, so to speak can be made and the means of attachment decided upon, this decision being governed by all the principles applicable to the same.

The next physiological consideration is that of devitalization. This one has caused enough discussion to fill volumes. Some have argued that "the tooth should *always* be devitalized when used as an abutment and others have said that "the nerve should *never* be destroyed." The arguments are too well known to be repeated here, but it deserves some recognition. To the mind of the writer, however, this question is one of minor importance and might also be added, deserves very little consideration when compared with other facts. In the posterior part of the mouth the mechanical and anatomical principles are those which demand chief attention. If the vitality of the tooth in any way interferes with the proper application of these principles it should most certainly be destroyed, if not, there is no reason why it should not be retained. In the anterior part of the mouth the esthetic, mechanical and anatomical are of chief importance and just as in the posterior part if these are in any way impaired, destroy, and if not retain the vitality of the tooth.

Taking up the remaining requirements, the mechanical, anatomical and esthetic, this paper will consider them together, as applied in the posterior and anterior part of the mouth.

The Mechanical ranks first in importance in the posterior and probably second in the anterior part of the mouth. In the posterior region there is a tooth which has one of the strongest physiological attachments to the jaw and from its situation the operator is enabled to construct an attachment which can be made without a great sacrifice of tooth substance for the sake of appearances. The telescopic crown has been considered as being nearer a universal method for such location than any other and deservedly so, because not only can it be made to possess inherent strength but the abutment itself can be left more nearly in its normal condition than by any other principle.

The first that particularly attracts our attention in this connection is the preparation of the abutment and not only for the crown alone, but as the anterior abutment is usually one which is not so easily formable it is necessary that these posterior abutments should assume most of the burden of parallellism between the two.

What is the correct preparation for a telescopic crown? In the first place only as much tooth substance should be taken from the top of the tooth as will be necessary for a strong occlusal surface to crown. Then the walls should be ground down so as to make the greater circumference just beneath the gum line, then it may be necessary to grind more for establishing the parallel lines, as before mentioned. This is all that should influence the operator, true it is sometimes necessary to remove the enamel but this is not expedient in the majority of cases; never in the lymphatic type, more frequently in the sanguine and still more in the bilious and nervous. Too much stress can not be laid upon the technic in the preparation of the abutment for a telescopic crown. The operator should never forget that he is working upon extremely sensitive tissue; the fifth nerve is by far the most susceptible of all cranial nerves; there is a limit to its irritability therefor be as expeditious and careful as possible. In using stones for grinding revolve away from the tooth so as to push the gum back rather than the hammering of it against the tooth as you do by the reverse; cross cut enamel burs work faster in some locations; small stones mounted on mandrels for the countra or right angle have their place; excising forceps can also be used quite frequently. Although each operator has his own method which he can use best, still the above suggestions should always be followed.

Taking up the consideration of the telescopic crown the anatomical requirement will be considered with the mechanical, the esthetic is of no consequence in this location. There have been two methods of construction up until a recent time. (1) Sectional (natural bite preferred) and (2) Seamless; but lately has been added the cast method which will be considered in another place in this paper. The essential requirements of a completed crown are much the same as that given for bridges: It should be made of sufficiently high karat to withstand any chemical action which might take place in the mouth; and of a gauge necessary to stand any stress put upon it; it should be a perfect fit to the abutment, passing a uniform distance beneath the gum line without irritation; it should restore the point of contact with approximating tooth it presents and be contoured to typical shape; it should have perfect occlusion, with cusps prominent enough to aid in the act of mastication but not so much as to interfere with the movements of the mandible. It should be in perfect alignment; and, last and most important, when placed on the abutment, it should receive the stress of mastication in such direction as to assist rather than be a menace to the integrity of the bridge.

In taking up the anterior part of the mouth, as has been stated, it is the opinion of the writer that the esthetic is the important consideration, with the anatomical second and the mechanical third. This means, then, that the strength of attachment should be sacrificed for the sake of appearance; it does not mean, however, that the stability should be entirely overlooked.

Anterior attachments may be classified as (1) those to roots as band and dowel and (2) as those to the natural crown of tooth. In the latter the endeavor is made to obtain adequate support with the conservation of as much tooth as possible. Of the dowel crowns there are the band and dowel, the plate and dowel.

The advantages claimed for the first over the second are (1) Better sealing of the root (2) Protection of the root against fracture (3) Stronger attachment for bridges. The advantages of the plate crown over the band are: (1) More natural restoration possible. (2) The securing of normal condition of the gingiva. (3) Preservation of the continuity of the root. It is the opinion of the writer that the band and dowel is used much more frequently than indicated. The deleterious effects produced by irritation from the band alone are of more consequence than the extra strength of attachment gained.

In the technic of root preparation here again extreme care should be exercised; save the patient as much as possible. In the preparation for the band it is absolutely necessary to obtain correct form; there should be no undercuts at any point around the circumference of the root and this necessitates the removal of the enamel in nearly all cases. It should extend slightly beneath the gum line on the labial and leave as much as possible on the lingual so as not to interfere with the facing.

In the preparation for the plate crown, the saddle effect is the best form and that following cervical second.

Taking up the mechanical structure of the band and dowel it is the opinion of the writer that the band should be always made of iridio platinum of 34 or 36 gauge, and the top of the cap with platinum of the same gauge. The band should have a long lap and all soldering be done with pure gold and heated until the alloy is formed, to prevent future unsoldering. In this manner a band is made which will not stretch and if properly adapted will not allow any movement independent of the root and not afford much chance for irritation.

For the plate crown if the root is irregular, thin platinum should be used, about 34 gauge. In regular, with straight slants, platinum of 39 gauge is better. The iridio platinum dowel is the most desirable and should be made round and tapering. The prevention of the rotation of the crown on the root should not depend upon the shape of the dowel.

In the selection and adaptation of the facing remember that the esthetic is of the first importance; so do everything possible to make the tooth simulate nature; remember the natural curve at the cervical, let it follow the gingival line; keep the tooth in alignment both at neck and at the incisal edge; select the correct color and keep in mind the shading produced by different backings. It is the opinion of the writer that it is better to leave the incisal edge free rather than grind a bevel for a gold tip, this again on account of the esthetic effect.

It is the purpose of this paper to compare the ideal attachments of the old form to those which have been made possible through the principle of casting and which might, for the advantage in discussion, be termed "new form." The discussion which has gone before is, in a brief manner, a statement of the requirements of the gold form attachments. A careful examination of the latter shows that, although ideally conceived and perfectly executed, still they are far from being the thing sought. The telescopic crown will always require the destruction of the periphery of the abutment at the gingival line and there will always be a certain amount of irritation of soft tissues due to the presence of the gold band. The band and dowel, although probably the strongest of all crown anterior attachments, yet has its disadvantages, namely, irritation from band and difficulty in restoring the natural contour. The plate and dowel crown although it can be made more nearly perfect from the esthetic standpoint, yet it lacks strength, owing to the shortness of the dowel and lack of concomitant means of attachment.

It can readily be seen then that any step towards the preservation of the tooth in its natural contour of crown is one in the right direction, as has been inferred, no matter how much an artist the operator may be, he can never exactly restore the natural position; our own teeth in normal position and form are the best, so any attachment which tends to minimize the loss of tooth structure is a decidedly progressive act. Unfortunately the lack of ability and misconceived ideas have greatly prevented our advancement in this direction.

The development of the casting principle has, by the adaptation of the so-called "partial crown attachments" at last put into our hands the means to make a strong attachment and still retain the continuity of the natural tooth so desirable. In order to be perfectly understood a "partial crown attachment" is one which never encircles the tooth on the labial or buccal side by band but security of position is obtained by different mechanical means to the lingual or lingual-occlusal surfaces. These, in the past, have never been considered a very strong abutment-piece but have been used as an anterior support to a more firm posterior. They have also been considered as a more or less temporary procedure when compared with the well constructed full crown. Their successful application depends upon selective judgment, care in the preparation of the abutment and accuracy of construction and if these are carefully carried out to the mind of the writer, there is nothing better for attachments in locations which will be enumerated later. Even if considered as a *temporary procedure* it is warranted in many cases from the esthetic requirement alone.

If one will consider the attachments which have been and of course are still in use (which this paper has classed as old style attachments) the conservation of tooth structure has been deemed desirable and has in many ways been sought for. The open face crown is a monumental example. The only reason it gained such a place in our principles was because it could be used on a vital tooth and that more of the normal continuity of tooth structure could be retained. This attachment has probably been more discussed, and might be added *cussed,* than any other and not without cause, for it is probably the reason of more failures in bridge work than any other one method. To the mind of the writer, however, this was due more to misapplication and lack of care in construction than mistake in principle.

They should never be used on any superior teeth except the cuspids and on the cuspids and laterals of the inferior, yet in rare cases where vital teeth are desired it still has its use in these locations.

The writer wishes to present several locations where it is possible to retain the anatomical conformity of the tooth to a great extent and yet construct upon them both permanent and sufficiently strong attachments for any kind of bridge. This can be done by the "Inlay and Dowel," "Carmichael" and "Partial Telescopic." The development in the process of casting has made this both ideal and possible.

The Inlay and Dowel is a modification of the old "plate and pin" so-called. It is particularly adaptable on superior cuspids; it can also be used on superior laterals and inferior cuspids and bicuspids. The advantages in principle obtained in this abutment piece are strength of attachment through the long dowel and distribution of stress through a greater amount of tooth substance. The inlay itself should not be considered as giving any extra strength; it merely acts as a means of uniting the dummy to the attachment, although some slight assistance may be given by the correct preparation of the abutment, particularly on the lower bicuspids.

The construction is as follows: Take the tooth where it has its greatest use, perhaps as an example, superior cuspid. This tooth should be devitalized, the canal filled and the tooth ground to the correct form, which is as follows: The lingual surface should be ground to a sufficient depth to allow from 1-16 to ⅛ of an inch of depth to the inlay and yet accommodate the articulation. Grind to good definite edges allowing it to extend slightly over the mesial and distal surfaces to form saddle effect if possible, and carry the side to be soldered to the dummy to a point which will allow the protection of the cervical border of the inlay during the soldering process. A good thick incisal edge should be left intact. The canal is then reamed out to accommodate the dowel, which should be of iridio-platinum of 12 to 14 gauge, round, tapering to the end. The top portion can be flattened out, split or roughened to form good adhesion to the gold when cast around it. The portion of the canal near the surface is then enlarged to allow the inlay wax to surround the head of the dowel and extend slightly down the latter. The inlay wax is then forced into place; in case of the lower bicuspid the Ivory matrix is of greatest assistance here, keeping good definite edges. The dowel is then heated and forced down into the canal through the wax, cooled and trimmed to shape, invested and cast. The writer prefers to leave the head of the dowel projecting slightly above the wax, trimming down into the same around the dowel so as to facilitate in pulling from the tooth and also to flow solder around in soldering to the dummy.

After casting it can be fitted to the tooth, ground to articulation and it is ready for the bridge. This makes a very strong anterior attachment with the least amount in loss of tooth structure. The abutment is also stronger as the stress of mastication is spread over a greater length of tooth and there is nothing to interfere with the normal relation at the gingival attachment. The connection with the dummy can also be made at the point of contact, thus restoring the interproximal space.

The Carmichael principle is familiar to all. It is particularly suggested on superior bicuspids. The advantage of the cast method is in the ease of construction and accuracy of the fit obtained. It is possible also to restore the full contour. The best preparation of the tooth is to first cut away the lingual cusp, then taper this portion, making a definite edge just below the point of contact; then the three grooves, one on the mesial, another on the distal and a third on the occlusal formed for retention. This can be done with small stones and cross cut fissure burs. It should be so shaped that wax will easily draw from the same. If greater strength of attachment is necessary, the tooth can be devitalized, the canals filled and a dowel adapted. The wax model is obtained by taking the impression over the tooth as prepared alone, or with the dowel, invested and cast; then fitted to the tooth when it is ready for the bridge. One is greatly assisted in the taking of the wax impression here by the use of the before mentioned Ivory matrix. Drawing it around the tooth and pulling together on the buccal side.

Taking up the last, the cast "partial telescopic" as it might be termed, the writer believes it has a great future. In this the telescopic principle is retained and, in short, all the advantages of the shell crown are retained but *none* of its disadvantages. It may also be made in some cases without devitalization of the tooth.

The preparation of the abutment is as follows: The occlusal surface of the tooth should be ground off to allow for the restoration of the same in the crown. This remaining portion should then be tapered to a well defined edge in the tooth's substance on the mesial, distal, buccal and lingual, being slightly below the point of contact on the two former surfaces and as low on the two latter as is necessary to strengthen the attachment. After smoothing up it is then ready for the crown. Now construct the band of pure gold of 30 gauge by sweating together, fit to the tooth, allowing it to project slightly above so there will be a surface to which the wax can adhere. The inlay is then put in place, having the tooth slightly oiled, the band dry and warm and the bite then taken. This is then taken from the tooth, carved up and prepared for casting, then invested and cast and finally fitted to the tooth when it is ready for the bridge.

This can be done in middle age or elderly people qutie frequently without devitalization of the tooth. The writer prefers, however, where devitalization is necessary, to use a dowel in conjunction with the crown, extending it into the lingual canal of the upper molars and distal of the lower thereby gaining extra strength of attachment and stability to the crown.

This paper has not entered into a discussion of inlays as a means of attachment as the writer deems them far inferior to these mentioned here. He does not consider them of any value whatever except as a seat for an occlusal rest.

